# Promoting Sleep Health Among Families of Young Children in Head Start: Protocol for a Social-Ecological Approach

**DOI:** 10.5888/pcd13.160144

**Published:** 2016-09-01

**Authors:** Karen A. Bonuck, Arthur Blank, Barbara True-Felt, Ronald Chervin

**Affiliations:** Author Affiliations: Arthur Blank, Albert Einstein College of Medicine, Bronx, New York; Barbara True-Felt, Ronald Chervin, University of Michigan, Ann Arbor, Michigan.

## Abstract

Inadequate or poor quality sleep in early childhood impairs social–emotional and cognitive function via effects on the developing brain and increases obesity risk via hormonal and endocrine effects. The prevalence of short sleep duration, behavioral sleep problems, and sleep-disordered breathing among children aged 3 to 5 years is 20% to 50%. Healthy sleep habits increase sleep duration and prevent behavioral sleep problems. Awareness of sleep-disordered breathing symptoms leads to its timely treatment. We designed a study that aims to empower families whose children are in early childhood programs with the knowledge and skills needed to obtain healthy sleep and to recognize a sleep problem. We used the social–ecological framework to guide individual, interpersonal, organizational, community, and policy interventions. This study builds on the Sweet Dreamzzz, Inc, Early Childhood Sleep Education Program (ECSEP) in Head Start. A stepped-wedge–cluster randomized trial will test effects on child, parent, and classroom outcomes; a policy evaluation will assess the impact of knowledge-translation strategies. The study has 3 aims. The first is to adapt educational materials into multimedia formats and build the capacity of Head Start agencies to implement the study. The second aim is to enroll 540 parent–child dyads in a primary prevention trial of sleep health promotion in Head Start and to analyze effects on children’s sleep duration (primary outcome); parents’ knowledge, attitudes, self-efficacy, and behavior; and children’s sleep difficulties. The third aim is to conduct a secondary prevention feasibility study of screening and guidance for sleep problems. Secondary outcomes are changes in classroom behaviors and policies. Integrating sleep health literacy into early childhood programs could affect the life-course development of millions of children.

## Sleep as a Health Behavior and Rationale for a Study Designed to Improve Sleep Health Literacy

Despite the importance of adequate sleep, public health promotion of sleep as a health-related behavior is minimal (1). Early childhood is a critical time in which the foundations of life-long health are built. Healthy sleep is a key but often neglected building block. During early childhood, sleep habits are established, and interventions are often more feasible and effective than those undertaken later in life.

The most modifiable, prevalent, and consequential sleep problems in early childhood are insufficient sleep, behavioral sleep problems, and sleep-disordered breathing. The latter 2 problems peak during the preschool years (children aged 3–5 y) and can contribute to insufficient sleep. The National Sleep Foundation recommends that children aged 3 to 5 years sleep 10 to 13 hours per day (2), which the American Academy of Sleep Medicine recommends should include naps (3). An estimated 25% to 50% of preschool children do not sleep enough (4). Behavioral sleep problems are problems with falling asleep or staying asleep (eg, bedtime resistance, night wakings) that correspond to diagnostic classifications of “sleep onset association,” “limit setting,” or “combined” types of behavioral insomnia (5). Approximately 25% of preschool children have behavioral sleep problems (6). From 1% to 20% of preschool-aged children experience sleep-disordered breathing, which spans a continuum from snoring to obstructive sleep apnea (7).

Despite some differences in etiology, these sleep problems affect areas of the brain and can adversely affect cognitive and social development (8). Irregular bedtimes for children aged 3 to 7 years can lead to poor academic and spatial skills (9) and problem behaviors (10). Likewise, insufficient sleep for children aged 2 through 6 years affects cognitive function (11) and behavior (12) and may cause hyperactivity (13). In a population-based study of 11,000 children, sleep problems before 5 years of age increased the likelihood of requiring special education at 8 years (14). The sleep problems of young children are also linked to obesity. Short sleep duration and sleep-disordered breathing before age 5 years independently increased the likelihood of being overweight at age 15 (15). 

Sleep problems of young children can be prevented. Good sleep hygiene serves to entrain circadian rhythms, condition behavior, and reduce stimulation. For young children, this means having a regular bedtime (before 9 PM) and bedtime routine, falling asleep without the presence of a parent, and eliminating evening screen time. Poor sleep hygiene is linked to high rates of insufficient sleep and behavioral sleep problems among preschoolers (16–18). Behavioral strategies to improve sleep hygiene promote healthy sleep (19) and address sleep problems (20). They are most effective for young children (21) because they target parent–child interactions that contribute to the problem (22). 

Finally, disparities in sleep health among racial/ethnic populations are substantial but modifiable and include differences in sleep duration and sleep hygiene. For example, interactive bedtime routines are less common among low-income and racial/ethnic minority families than among their socioeconomically advantaged, non-racial/ethnic–minority counterparts (23,24), whereas parent–child co-sleeping is more common. Rates of sleep-disordered breathing also are higher among racial/ethnic minority and low-income children, and these children are least likely to receive treatment for the disorder (25). Cultural values influence sleep practices; however, sleep health disparities could be reduced by increasing parents’ knowledge and skills about how to obtain healthy sleep for their children.

We designed a multilevel study aimed at increasing sleep health literacy — the knowledge and self-management skills needed to obtain healthy sleep and to recognize signs of a sleep problem — among families with children in Head Start, a federal program of early childhood care and education for low-income families that serves mainly racial/ethnic minorities. The study has 3 components: 1) a pre-implementation phase in which we adapt existing materials for the study and build the capacity of Head Start agencies to implement the study, 2) a primary prevention trial of sleep health promotion activities in early childhood care and education, and 3) a secondary prevention feasibility study of screening and guidance for sleep problems.

## Study Design

Our study design was guided by the social–ecological framework (26), which specifies 5 levels in the determinants of health. Our study encompasses all 5 levels: individual, interpersonal, organizational, community and policy (27) (Table). Another framework for this study is health literacy, defined as the capacity to obtain, process, and understand information needed to make health-related decisions. Our study aligns with the national health literacy goal to “embed accurate, accessible and actionable health information in all early childhood programs, such as Head Start” (28).

**Table:** Interventions in the Head Start Program to Promote Sleep Health, by the Five Levels of the Social–Ecological Model

Level	Intervention Strategy	Implemented by	Goal
Individual	The program does not intervene at the individual level; only outcomes are available at this level.		
Interpersonal	Early Childhood Sleep Education Program (ECSEP)^a^	Head Start agency	Increase sleep duration
	Family 1-to-1 sleep health contacts^b^	Head Start agency	Amplify ECSEP messages
Organization	Media campaign	Research team	Amplify ECSEP messages
	Agency education	Research team	Support staff engagement
Community	Health Services Advisory Councils^c^	Research team	Support local engagement
	Local pediatricians	Research team	Support feasibility study
	Local sleep medicine specialists	Research team	Support feasibility study
Policy	Knowledge-translation strategies	Research team	Sustainability

a The ECSEP educates Head Start staff, parents, and children about healthy sleep (19).

b These are one-to-one contacts between Head Start staff members and families.

c Health Services Advisory Councils comprise parents, staff, and local health care professionals.

### Pre-implementation: adapting materials to new formats and building agency and community capacity

To prepare for the primary prevention trial we will adapt evidence-based materials into new formats and build capacity of Head Start agencies and communities to implement interventions. We will incorporate into our trial the Early Childhood Sleep Education Program (ECSEP) developed by Sweet Dreamzzz, Inc; its core content is interactive, skills-based, and actionable. All new materials developed will use the health literacy principles of clear communication. Using the Suitability Assessment of Materials tool, we will rate new materials on content (eg, applied vs factual), literacy demand (a composite of such elements as reading grade level, active voice, common vocabulary words, content, and road signs), graphics, layout and typography, learning stimulation and motivation, and cultural appropriateness (29). Parental materials will be translated into Spanish, back translated, and finalized at a 4th- or 5th-grade reading level.

We will develop ECSEP-based print and video materials that reinforce and amplify ECSEP content: a tip sheet on healthy sleep to engage families at enrollment (30), an educational flipchart for one-to-one contacts between Head Start staff members and families (the Family 1-to-1 intervention), posters for Head Start, and a brochure for parents. The brochure will include a tear-off section for the family to write in 1 or 2 attainable goals. Finally, we will produce 5-minute videos in English and Spanish about healthy sleep for families.

We will also prepare materials for the secondary-prevention feasibility study of screening and guidance for sleep problems. For behavioral sleep problems, we will develop an evidence-based educational flipchart and brochure for parents (31). Master’s-level Head Start managers will use these materials to guide conversation and goal setting with parents. For sleep-disordered breathing, our team’s sleep medicine experts will develop scripts for managers to use when discussing screening results, a referral packet for parents to bring to their child’s doctor, and education packets for general pediatric health care providers. Provider packets will include electronic access to learning modules, actionable steps for assessing and managing sleep problems, and contacts for local sleep medicine specialists.

To build agency capacity, we will apply a pioneering implementation platform. The Health Care Institute at the University of California, Los Angeles, Anderson School of Management uses management principles that engage and motivate Head Start staff and parents to deliver health literacy programs (32). The Health Care Institute’s programs have reached 100,000 Head Start families. Following the Institute’s model, agencies formed leadership teams that will conduct virtual meetings to review materials and tailor protocols to local need. Teams will attend a 2-day training summit in February 2018 where they will practice using tools and role playing scripts and administering and scoring study measures. During a test phase they will conduct mock data collection activities and referrals.

### A stepped-wedge–cluster randomized controlled trial (RCT) of educational interventions

We will conduct a stepped-wedge–cluster RCT of the ECSEP, Family 1-to-1 intervention, and media campaign (Figure). The stepped wedge is a type of cluster-randomized trial, in which all clusters begin as controls and then cross over (permanently) to the intervention at randomly assigned time points. This design is ideal when individual-level randomization is impractical, interventions are multilevel, and stakeholders view interventions as having benefit (33). Although Head Start agencies comprise one or more sites, training and protocols are implemented agency-wide. Thus, we will randomize at the agency level. The stepped wedge design’s staggered implementation controls for time trends and has logistical benefits for multilevel interventions. Finally, agencies are eager to implement the interventions and want to disseminate them to all their sites.

**Figure:**
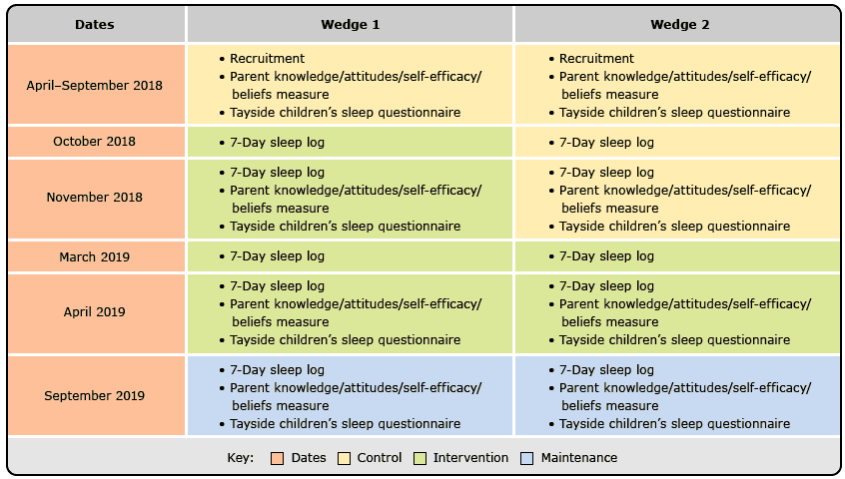
Individual-level outcomes in the stepped-wedge–cluster design of the randomized control trial. **Table:** 

Dates	Wedge 1	Wedge 2
April–September 2018	Control: Recruitment, Parent knowledge/attitudes/self-efficacy/beliefs measure, Tayside children’s sleep questionnaire	Control: Recruitment, Parent knowledge/attitudes/self-efficacy/beliefs measure, Tayside children’s sleep questionnaire
October 2018	Intervention: 7-Day sleep log	Control: 7-Day sleep log
November 2018	Intervention: 7-Day sleep log, Parent knowledge/attitudes/self-efficacy/beliefs measure, Tayside children’s sleep questionnaire	Control: 7-Day sleep log, Parent knowledge/attitudes/self-efficacy/beliefs measure, Tayside children’s sleep questionnaire
March 2019	Intervention: 7-Day sleep log	Intervention: 7-Day sleep log
April 2019	Intervention: 7-Day sleep log, Parent knowledge/attitudes/self-efficacy/beliefs measure, Tayside children’s sleep questionnaire	Intervention: 7-Day sleep log, Parent knowledge/attitudes/self-efficacy/beliefs measure, Tayside children’s sleep questionnaire
September 2019	Maintenance: 7-Day sleep log, Parent knowledge/attitudes/self-efficacy/beliefs measure, Tayside children’s sleep questionnaire	Maintenance: 7-Day sleep log, Parent knowledge/attitudes/self-efficacy/beliefs measure, Tayside children’s sleep questionnaire

At baseline, beginning in April 2018, we will enroll 540 parent–child dyads from 7 agencies (Box). All Head Start agencies will begin as controls, and then cross over (permanently) to the intervention at 2 randomly assigned times or “wedges.” As part of the stepped-wedge design, data are collected at baseline as well as before and after each cluster crosses over to the intervention. In our study, agencies and their sites (n = 21) will cross over to the intervention in 2 wedges (3 or 4 agencies per wedge) during the 2018–2019 school year. Data will be collected at baseline, before and after ECSEP interventions in each wedge, and at 1-year follow-up.

**Box. Project Timeline for Sleep Health Promotion in Head Start Program**

**Year 1**

Adapt and develop materials.

Build agency and community capacity.

Pilot test sleep health and problem protocols.

Engage with partners on policy (ongoing).

**Year 2**

Adapt and develop materials.

Build agency and community capacity.

Pilot test sleep health and problem protocols.

Assess policy climate.

**Year 3**

Participate in Health Care Institute summit.

Pilot test procedures.

Enroll dyads and collect baseline data (beginning in April 2018).

Engage in policy dialogues.

**Year 4**

Enroll dyads and collect baseline data (through September 2018).

Train and implement Wedge 1 (August 2018–October 2018).

Train and implement Wedge 2 (March 2019–October 2019).

Conduct feasibility study.

Continue dialogue and partner activity on policy.

**Year 5**

Final 12-month follow-up (September 2019).

Analyze data.

Finalize manuscripts.

Assess policy climate and impact of policy.

Agency staff members will recruit English- and Spanish-speaking parents of children aged 3 years. Enrolling only 3-year-olds enables us to control for normal shifts in sleep patterns. Dyads that do not enroll will still be exposed to school-wide interventions. Agency staff members will enroll dyads when parents register their child in Head Start for the school year. We originally planned for staff members to collect data from parents at daily drop-off or pick-up. After discussion with our rural collaborators we learned that long bus rides negate this option; we are discussing alternatives, such as surveys sent home in backpacks and follow-up telephone calls.

#### Five levels of the social–ecological framework applied to the RCT

**Individual-level outcome measures and hypotheses.** The RCT’s primary outcome, which we used to determine sample size, is weeknight sleep duration based on 7-day sleep logs. Agency staff members will distribute and collect the logs from parent participants 1 week before and after each wedge crossover and at final follow-up (5 data points). We will test the hypothesis that children in the intervention group will sleep longer than children in the control group; we anticipate a difference of at least 30 minutes through the end of wedge 2 and a 15-minute difference at final follow-up. Analyses will be based on weeknight (Sunday through Thursday) sleep measured in minutes. Weeknight sleep is less variable than weekend night sleep, and these data are pertinent for school. Hypothesis tests will rely on a linear mixed model (34). Random effects at the agency and dyad level will be incorporated.

To assess sleep difficulties, we will administer to parents the Tayside children’s sleep questionnaire, a simple tool that taps into ECSEP content (ie, night-waking, bedtime resistance) among children aged 1 to 5 years. Nine items referring to the previous 3 months are scored on a 5-point scale (from zero to 4). A score of 8/36 indicates a mild or moderate settling difficulty. The last (yes/no) item asks the parent whether the child has a sleep problem (35). This valid and reliable tool (36,37) was piloted with parents of Head Start children (38). We will test the hypotheses that the intervention group, compared with the control group, will have lower mean scores, less likelihood of mild or moderate sleep difficulties, and lower rates of sleep problems.

To measure parent knowledge, attitudes, self-efficacy, and beliefs (KASB), we will administer the parent KASB measure used in the previous trial of ECSEP (19) to reflect the content in the parent workshop. Twelve knowledge items refer to bedtime routines such as going to bed at the same time each night. Five attitude items ask whether better sleep at night would, for example, make their child “less moody the next day.” Seven self-efficacy items elicit confidence in, for example, the ability to “work toward a goal of 8 PM for my child’s bedtime.” Two belief items ask about the value of a regular bedtime and bedtime routine. A multiple-choice item asks about how much sleep a preschooler needs. Analyses will test our hypotheses that the intervention group, compared with the control group, will have higher total KASB scores and higher KASB domain scores. We will conduct analyses before and after the ECSEP in each agency.

**Interpersonal-level interventions.** ECSEP, the core intervention, integrates seamlessly into Head Start’s training schedule and educational model. Teachers are trained to teach a 2-week (40 min/d) curriculum that aligns with their daily routine and teaching standards. Children learn through stories about their need for sleep and a bedtime routine. Children take home a teddy bear, bedtime routine sticker chart, and book. Parent workshops are held 1 week before the classroom lessons so parents are primed to use the sticker chart and other bedtime aids.

Family 1-to-1 sleep health contacts will amplify the ECSEP’s messages. Enrolled parents will receive at least 1 such contact as part of the four Family 1-to-1 contacts mandated by Head Start. During the pre-implementation phase, each agency will determine the best-qualified staff member and context for these settings.

Sweet Dreamzzz will conduct all training sessions on ECSEP materials. Teachers will receive a curriculum guide and all supplemental materials. Head Start managers designated to lead the parent workshop will observe its delivery by Sweet Dreamzzz, participate in a train-the-trainer session, and receive all training and reference materials. Sweet Dreamzzz staff members will also teach the designated Head Start manager how to use the ECSEP-based educational flipchart.

**Organizational-level interventions and outcomes. **We will raise awareness of sleep health among agency staff members and families through agency-wide training and a communications campaign. The agency training will focus on the relationship between sleep problems and school readiness, adult sleep health tips, and the study’s logistics. We will launch a communications campaign with posters, brochures, and a video when agencies cross over to the intervention. Posters and brochures will be placed in public areas at sites, and brochures will be sent home in students’ backpacks. The family video will air on televisions, if available, at each site.

To assess outcomes we will collect measures of classroom climate, as reflected by teacher–child interactions. The Classroom Assessment Scoring System (CLASS) is an evidence-based tool that uses certified observer ratings. We will analyze CLASS data for positive climate, negative climate, and behavior management. We hypothesize that differences will exist between intervention and control scores at the classroom, site, and agency levels. We will conduct analyses before and after the ECSEP in each agency.

**Community-level interventions.** To promote community awareness and to support the primary and prevention studies, we will engage each agency’s Health Services Advisory Council (HSAC). HSACs comprise parents, staff, and local health care professionals. Researchers will present information at each HSAC meeting and invite its members to the parent workshop. To promote awareness of the study among local health care providers and increase their capacity to respond to parents’ concerns, we will email the outreach and education packets to local pediatricians and sleep medicine specialists. We anticipate enabling pediatricians’ access to online resources. In line with the study’s health literacy approach, resources that provide synthesized evidence and actionable steps will be prioritized. Reaching out to local sleep medicine specialists will alert them to potential referrals.

**Policy interventions and outcomes.** To extend and sustain the study’s impact we will implement a knowledge-translation strategy (39). In 2016, we conducted web-based and telephone-based environmental scans of stakeholders. The scan of early childhood care and education websites will detect content on sleep health and sleep problems. Telephone interviews will assess understanding and prioritization of engagement in sleep health literacy. We will engage a partnership group of policy makers and stakeholders to facilitate improvement of early childhood care and education guidelines and policy. To assess policy impact, we will conduct a baseline scan in year 1 and a follow-up scan in year 5.

#### Statistical analysis

Sleep duration is the primary outcome. In a previous RCT, participants in the ECSEP group slept longer (30 min/night) (19) than participants in the control group, a clinically significant effect. A sample size of 173 will provide 90% power to detect a difference as small as 15 minutes between the intervention and control groups (*P* < .05, 2-tail). For parent KASB, a secondary outcome, a sample size of 450 will provide more than 97% power to detect a moderate effect size (Cohen *d* = 0.3) for each of the knowledge, attitude, and belief scales. We will enroll 540 dyads and anticipate having 430 dyads at 12-month follow-up.

We will assess fidelity to the ECSEP protocol by observing randomly selected classrooms. For process measures, agencies will track the number of children and parents exposed to the ECSEP and the number of families contacted and screened for the sleep problem feasibility trial. For parent feedback, we will conduct semistructured telephone interviews with a random 15% subsample. To obtain staff feedback, we will interview staff members who were Family 1-to-1 sleep health contacts and who implemented the sleep problem protocol.

### Secondary prevention feasibility study

We will assess the feasibility of implementing a sleep problem screening, education, and referral program. This program would be akin to the role of Head Start staff members who conduct nutritional and developmental screening and offer first-line guidance or referral. The lack of any previous relevant data renders this study a feasibility study. A random 15% subsample from each agency will be selected for screening. The Children’s Sleep Habits Questionnaire (CSHQ) will screen for behavioral sleep problems, and the Pediatric Sleep Questionnaire Sleep-Related Breathing Disorders (PSQ-SRBD) scale will screen for sleep-disordered breathing. Both are valid and reliable for children aged 3 to 5 years (37,40,41). The CSHQ screens for multidimensional sleep problems (42,43). The PSQ–SRBD scale was validated against polysomnography, is available in several languages, and is used in research and clinical settings (41).

A designated master’s-level Head Start manager will be trained via webinar. Scripts will guide dialogues with parents about screening results. Managers will provide referral packets to participants with sleep-disordered breathing. For behavioral sleep problems, managers will use the behavioral sleep problem flipchart to tailor a family action plan. If needed, referrals will be made to the child’s physician or a sleep medicine specialist. We will collect data on total scores, domain scores, and the number of children who are identified by the screening process as having behavioral sleep problems or sleep-disordered breathing. For sleep-disordered breathing, we will calculate the proportion referred for medical evaluation.

## Conclusion

This article described a protocol for promoting healthy sleep in Head Start, the federal child development program for low-income children and their families. The study’s social-ecological framework was first formulated by Urie Bronfenbrenner (26), an architect of the Head Start program. Early childhood is the foundation for lifelong health. Education is a determinant of health across the lifespan; thus, linking early childhood programs and policies to health outcomes is critical. Integrating sleep health literacy into early childhood programs could affect the life-course development of millions of children.
